# Ductal carcinoma in situ: a risk prediction model for the underestimation of invasive breast cancer

**DOI:** 10.1038/s41523-021-00364-z

**Published:** 2022-01-14

**Authors:** Ko Woon Park, Seon Woo Kim, Heewon Han, Minsu Park, Boo-Kyung Han, Eun Young Ko, Ji Soo Choi, Eun Yoon Cho, Soo Youn Cho, Eun Sook Ko

**Affiliations:** 1 Department of Radiology, Samsung Medical Center, Sungkyunkwan University School of Medicine, Seoul, Republic of Korea; 2 Statistics and Data Center, Research Institute for Future Medicine, Samsung Medical Center, Seoul, Republic of Korea; 3 Department of Information and Statistics, Chungnam National University, Daejeon, Republic of Korea; 4 Department of Pathology and Translational Genomics, Samsung Medical Center, Sungkyunkwan University School of Medicine, Seoul, Republic of Korea

**Keywords:** Cancer imaging, Breast cancer

## Abstract

Patients with a biopsy diagnosis of ductal carcinoma in situ (DCIS) may be diagnosed with invasive breast cancer after excision. We evaluated the preoperative clinical and imaging predictors of DCIS that were associated with an upgrade to invasive carcinoma on final pathology and also compared the diagnostic performance of various statistical models. We reviewed the medical records; including mammography, ultrasound (US), and magnetic resonance imaging (MRI) findings; of 644 patients who were preoperatively diagnosed with DCIS and who underwent surgery between January 2012 and September 2018. Logistic regression and three machine learning methods were applied to predict DCIS underestimation. Among 644 DCIS biopsies, 161 (25%) underestimated invasive breast cancers. In multivariable analysis, suspicious axillary lymph nodes (LNs) on US (odds ratio [OR], 12.16; 95% confidence interval [CI], 4.94–29.95; *P* < 0.001) and high nuclear grade (OR, 1.90; 95% CI, 1.24–2.91; *P* = 0.003) were associated with underestimation. Cases with biopsy performed using vacuum-assisted biopsy (VAB) (OR, 0.42; 95% CI, 0.27–0.65; *P* < 0.001) and lesion size <2 cm on mammography (OR, 0.45; 95% CI, 0.22–0.90; *P* = 0.021) and MRI (OR, 0.29; 95% CI, 0.09–0.94; *P* = 0.037) were less likely to be upgraded. No significant differences in performance were observed between logistic regression and machine learning models. Our results suggest that biopsy device, high nuclear grade, presence of suspicious axillary LN on US, and lesion size on mammography or MRI were independent predictors of DCIS underestimation.

## Introduction

Ductal carcinoma in situ (DCIS) is a preinvasive or noninvasive breast cancer defined as the proliferation of neoplastic cells within the mammary ducts without invasion into the surrounding tissue^1^. DCIS accounts for almost 30% of newly diagnosed breast cancers^2^. DCIS underestimation is defined as the failure to detect invasive cancer in a preoperative biopsy, with the actual diagnosis becoming evident only after a pathological examination of the surgical specimen^3^. The reported risk of underestimation varies from 14 to 43%^4,5^, with one meta-analysis estimating the risk at 25.9%^6^. The standard treatment for patients diagnosed with DCIS is wide local excision with radiation or mastectomy^7,8^. However, due to concerns regarding DCIS underestimation, routine sentinel lymph node biopsy (SLNB) may be necessary in patients with DCIS diagnosed by core needle biopsy (CNB)^9,10^. Unfortunately, axillary dissection is often accompanied by complications such as pain, numbness, and arm swelling^11^. Therefore, the preoperative prediction of upgraded diagnosis to invasive cancer could avoid unnecessary axillary surgery, including SLNB.

Studies have attempted to identify the risk factors for underestimation, including nuclear grade and radiological findings such as lesion size on imaging, mass on mammography or ultrasound (US), and final Breast Imaging Reporting and Data System (BI-RADS) assessment categories^2,4–6,10,12–15^. Previous studies have also revealed an association between the risk of underestimation and factors such as age, palpability, histologic suspicion of invasion, imaging guidance method, biopsy device, and other factors. Although several papers have analyzed or mentioned all three imaging findings (mammography, ultrasound [US], and magnetic resonance imaging [MRI]), they didn’t describe a prediction model or just evaluated limited imaging findings^15–17^. To our knowledge, there is no study for evaluating prediction model using all imaging findings (mammography, US, and MRI)

Machine learning (ML) is a computational method capable of learning to improve the performance of a task based on previous experience. The ML field is closely related to pattern recognition and statistical inference and has been applied to problems across many fields, including bioinformatics^18^. ML overcomes or reduces the impact of the limitations of commonly used statistical techniques, which usually consider a limited finite set of hypotheses in their evaluations. However, ML approaches generate models for prediction by extensively searching the model and parameter space; thus, these approaches have been adopted for predictive modeling and decision-making in biomedicine^19^. However, there are few reports on the use of ML techniques for the prediction of DCIS underestimation or examining the potential improvement in prediction performance using ML^11^.

Therefore, the present study aimed to identify clinicopathologic and imaging features that predicted an upgrade of DCIS to invasive carcinoma on final pathologic diagnosis and to compare the diagnostic performance of various statistical models, including ML techniques.

## Results

### Factors associated with DCIS underestimation

**Fig. 1:**
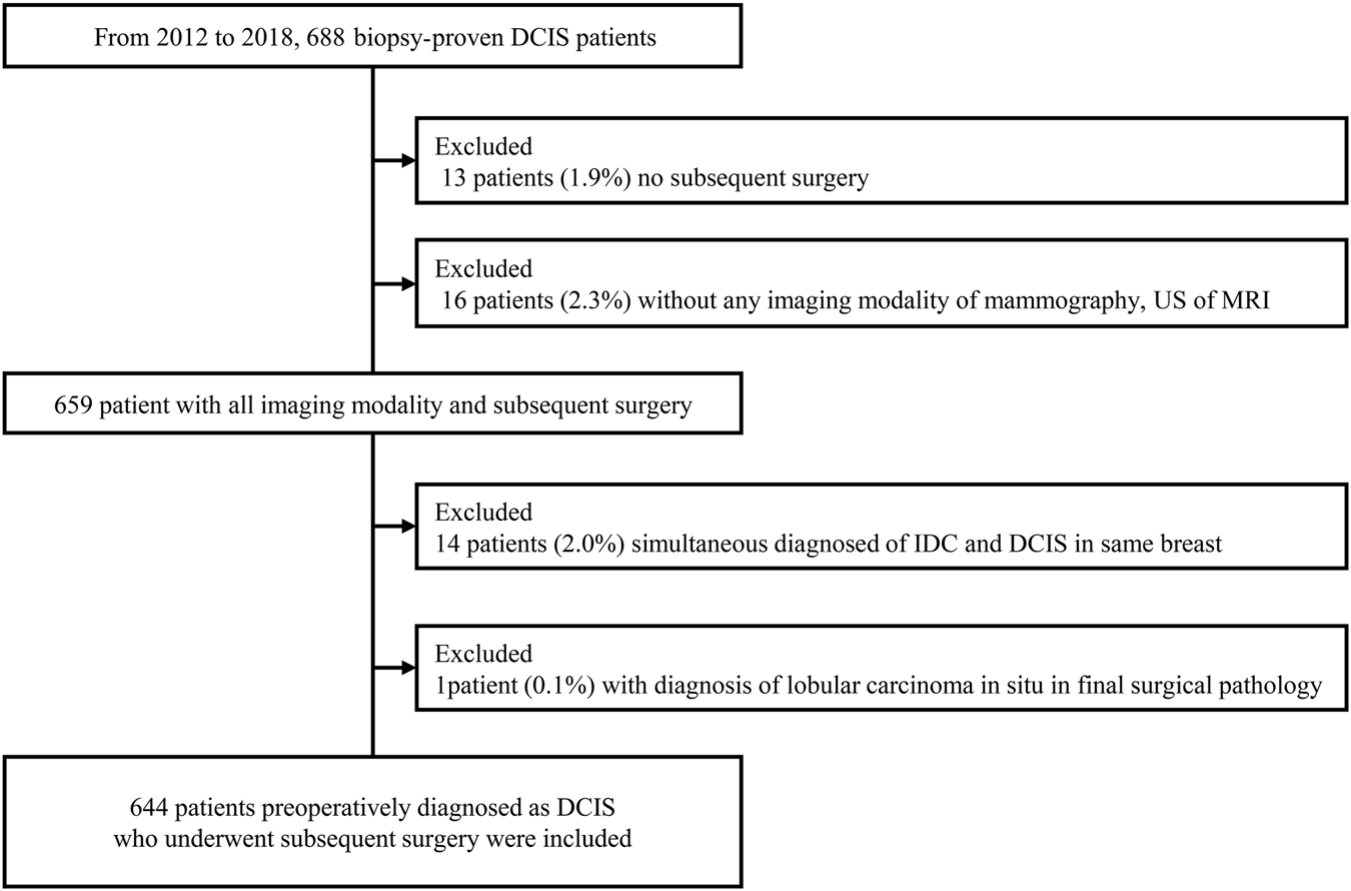
Flow chart of the study population. After we reviewed the biopsy database for biopsy-confirmed DCIS at our institution, we identified 688 biopsy-proven DCIS. This flowchart briefly presents how many patients were excldued and the reasons of exclusion.

Among 688 patients with biopsy-confirmed DCIS in our institution, we included 644 patients (mean age, 51.4; range, 22–87 years) who underwent subsequent surgery. Forty-four patients were excluded for the reasons shown in Fig. 1. Of the 644 DCIS lesions subjected to biopsy, 161 (25%) were underestimated invasive ductal carcinoma (IDC), including 73 identified as microinvasive cancers after surgery. The mean size of invasive cancer from surgical specimens was 3.89 mm (range, 0.01–35 mm, ±6.1 mm). Tables 1 and 2 show the patient characteristics and univariable analysis of the factors associated with the underestimation of invasive carcinoma. Palpability (*P* < 0.001) and lesion size >2 cm (*P* < 0.001) were significantly associated with histologic upgrade. Cases with mammography guidance (*P* < 0.001), vacuum-assisted biopsy (VAB) device (*P* < 0.001), thicker biopsy needle (*P* < 0.001), and a larger number of specimens (*P* = 0.001*)* were less likely to be underestimated. Pathologically, high nuclear grade (*P* < 0.001) and the presence of comedo necrosis (*P* < 0.001) were associated with underestimation. Imaging findings with cancer not visible on US or MRI resulted in significantly lower underestimation (*P* < 0.001 and *P* < 0.001, respectively). Mammographic findings indicated that combined mass/focal asymmetry with microcalcifications occurred significantly more frequently in the IDC group (*P* = 0.002). Fine linear/fine branching microcalcifications (*P* < 0.001) and linear/segmental distribution (*P* < 0.001) were also observed significantly more frequently in the IDC group. US findings showed that the presence of microcalcifications (*P* < 0.029), irregular mass shape (*P* < 0.001), suspicious axillary LN (*P* < 0.001), and high vascularity (*P* = 0.001) were significant indicators of underestimation MRI findings of irregular mass shape (*P* = 0.027), linear/segmental distribution of non-mass enhancement (NME) (*P* = 0.022), clustered ring enhancement pattern of NME (*P* = 0.003), and the presence of a washout pattern in the delayed phase of the time-intensity curve (*P* = 0.002) were significantly associated with underestimation.

**Table 1:** Patient characteristics and univariable analysis of factors associated with the underestimation of invasive carcinoma (clinicopathologic and mammographic findings).

Variable	DCIS (*n* = 483)	IDC (*n* = 161)	*P*	Odds Ratio (95% CI)	*P*
Age (years)	50.0 (45–58)	52.0 (47–58)	0.128		
Palpability			<0.001		
No	463 (95.9)	133 (82.6)		1	
Yes	20 (4.1)	28 (17.4)		4.87 (2.66–8.93)	<0.001
Guidance method			<0.001		
US	320 (66.3)	139 (86.3)		1	
Mammography	163 (33.7)	22 (13.7)		0.31 (0.19–0.51)	<0.001
Biopsy Device			<0.001		
CNB	225 (46.6)	111 (68.9)		1	
VAB	258 (53.4)	50 (31.1)		0.39 (0.27–0.57)	<0.001
Needle gauge, median (IQR)	13 (11–14)	14 (13–14)	<0.001		
Number of specimens, median (IQR)	6 (4–12)	5 (4–8)	0.001		
Nuclear grade			<0.001		
Low/Intermediate	366 (75.8)	82 (50.9)		1	
High	117 (24.2)	79 (49.1)		3.01 (2.08–4.37)	<0.001
Comedo necrosis			<0.001		
No	415 (85.9)	111 (68.9)		1	
Yes	68 (14.1)	50 (31.1)		2.75 (1.81–4.19)	<0.001
Mammography					
Density			0.833		
Fatty (grade A, B)	119 (24.6)	41 (25.5)		1	
Dense (grade C, D)	364 (75.4)	120 (74.5)		0.96 (0.64–1.44)	0.832
Mammography characteristics			0.002		0.002
Mass	30 (6.2)	9 (5.6)		1	
Focal asymmetry	11 (2.3)	4 (2.5)		1.21 (0.22–6.91)	>0.999
Calcifications	300 (62.1)	87 (54.0)		0.97 (0.36–2.62)	>0.999
Combined	50 (10.3)	38 (23.6)		2.53 (0.85–7.54)	0.133
Non- visible	92 (19.1)	23 (14.3)		0.83 (0.27–2.54)	>0.999
Dichotomized mammography characteristics			0.172		
Non- visible	92 (19.1)	23 (14.3)		1	
Visible	391 (80.9)	138 (85.7)		1.41 (0.86–2.32)	0.174
Mass shape (*N* = 94)			0.477		
Oval/round	21 (36.8)	11 (29.7)			
Irregular	36 (63.2)	26 (70.3)			
Mass margin (*N* = 94)			0.203		
Circumscribed/obscured	21 (36.8)	9 (24.3)			
Not circumscribed	36 (63.2)	28 (75.7)			
Calcification morphology (*N* = 476)			<0.001		
Fine linear/fine branching	80 (22.8)	50 (40.0)			
Fine pleomorphic	93 (26.5)	35 (28.0)			
Coarse heterogeneous/amorphous	178 (50.7)	40 (32.0)			
Calcification distribution (*N* = 476)			<0.001		
Linear/segmental	122 (34.8)	68 (54.4)			
Grouped/regional/diffuse	229 (65.2)	57 (45.6)			
Mammographic lesion size (cm)			<0.001		<0.001
≥2	173 (35.8)	107 (66.5)		1	
<2	218 (45.1)	31 (19.2)		0.40 (0.22–0.73)	0.001
Non- visible	92 (19.1)	23 (14.3)		0.23 (0.14–0.38)	<0.001

Numeric data are presented as medians, with the interquartile ranges in parentheses.

Non-numeric data are presented as the number of lesions (percentage).

*CI* confidence interval, *CNB* core needle biopsy, *VAB* vacuum-assisted biopsy, *LN* lymph node.

**Table 2:** Patient characteristics and univariable analysis of factors associated with the histopathologic upgrade to invasive carcinoma (US and MRI findings).

Variable	DCIS (*n* = 483)	IDC (*n* = 161)	*P*	Odds Ratio (95% CI)	*P*
US					
US characteristics			<0.001		<0.001
Mass	156 (32.3)	57 (35.4)		1	
Non-mass	178 (36.8)	86 (53.4)		0.33 (0.17–0.64)	<0.001
Non-visible	149 (30.9)	18 (11.2)		1.32 (0.84–2.08)	0.337
Dichotomized US characteristics			<0.001		
Non-visible	149 (30.9)	18 (11.2)		1	
Visible	334 (69.1)	143 (88.8)		3.54 (2.09–6.00)	<0.001
Calcifications on US (*N* = 477)			0.029		
No	148 (44.3)	48 (33.6)			
Yes	186 (55.7)	95 (66.4)			
Mass shape (*N* = 213)			<0.001		
Oval/round	64 (41.0)	7 (12.3)			
Irregular	92 (59.0)	50 (87.7)			
Mass margin (*N* = 213)			0.130		
Circumscribed	40 (25.6)	9 (15.8)			
Not circumscribed	116 (74.4)	48 (84.2)			
Mass orientation (*N* = 213)			0.821		
Parallel	135 (86.5)	50 (87.7)			
Nonparallel	21 (13.5)	7 (12.3)			
Echo pattern (*N* = 477)			0.136		
Isoechoic	91 (27.3)	26 (18.2)			
Hypoechoic	232 (69.5)	113 (79.0)			
Hyperechoic	1 (0.3)	0 (0)			
Complex echoic	10 (2.9)	4 (2.8)			
Posterior acoustic features (*N* = 477)			0.338		
Enhancement	19 (5.7)	7 (4.9)			
Shadowing	20 (6.0)	12 (8.4)			
Combined	22 (6.6)	15 (10.5)			
None	273 (81.7)	109 (76.2)			
Vascularity (*N* = 477)			0.001		
Low or none	98 (29.3)	20 (14.0)			
High	211 (63.2)	107 (74.8)			
Not available	25 (7.5)	16 (11.2)			
Suspicious axillary LN			<0.001		
No	476 (98.6)	127 (78.9)		1	
Yes	7 (1.4)	34 (21.1)		18.21(7.89–42.03)	<0.001
US lesion size (cm)			<0.001		<0.001
≥2	113 (23.4)	87 (54.0)		1	
<2	221 (45.8.)	56 (34.8)		0.16 (0.08–0.30)	<0.001
Non- visible	149 (30.8)	18 (11.2)		0.33 (0.21–0.52)	<0.001
MRI					
MRI characteristics			<0.001		0.001
Mass	142 (29.4)	52 (32.3)		1	
Non-mass enhancement	262 (54.2)	104 (64.6)		0.17 (0.06–0.52)	<0.001
Non-visible	79 (16.4)	5 (3.1)		1.08 (0.69–1.69)	>0.999
Dichotomized MRI characteristics			<0.001		
Non-visible	79 (16.4)	5 (3.1)		1	
Visible	404 (83.6)	156 (96.9)		6.10 (2.43–15.35)	<0.001
Mass shape (*N* = 194)			0.027		
Oval/round	34 (23.9)	5 (9.6)			
Irregular	108 (76.1)	47 (90.4)			
Mass margin (*N* = 194)			0.231		
Circumscribed	20 (14.1)	4 (7.7)			
Not circumscribed	122 (85.9)	48 (92.3)			
Internal enhancement of the mass (*N* = 194)			0.251		
Homogeneous	9 (6.3)	1 (1.9)			
Heterogeneous	121 (85.2)	47 (90.4)			
Rim-enhancement	12 (8.5)	3 (5.8)			
Dark internal septation	0 (0)	1 (1.9)			
Distribution of NME (*N* = 366)			0.022		
Linear/segmental	158 (60.3)	76 (73.1)			
Focal/regional/diffuse	104 (39.7)	28 (26.9)			
Internal enhancement of NME (*N* = 366)			0.003		
Homogeneous/heterogeneous/clumped	250 (95.4)	89 (85.6)			
Clustered ring	12 (4.6)	15 (14.4)			
Time–signal intensity curve (washout) (*N* = 560)			0.002		
No	180 (44.6)	47 (30.1)			
Yes	224 (55.4)	109 (69.9)			
MRI lesion size (cm)			<0.001		<0.001
≥2	226 (46.8)	126 (78.3)		1	
<2	178 (36.9)	30 (18.6)		0.11 (0.04–0.33)	<0.001
Non- visible	79 (16.3)	5 (3.1)		0.30 (0.18–0.50)	<0.001

Numeric data are presented as medians (interquartile ranges).

Non-numeric data are presented as the number of lesions (percentage).

*CI* confidence interval, *LN* lymph node, *MRI* magnetic resonance imaging, *NME* non-mass enhancement, *US* ultrasonography.

**Fig. 2:**
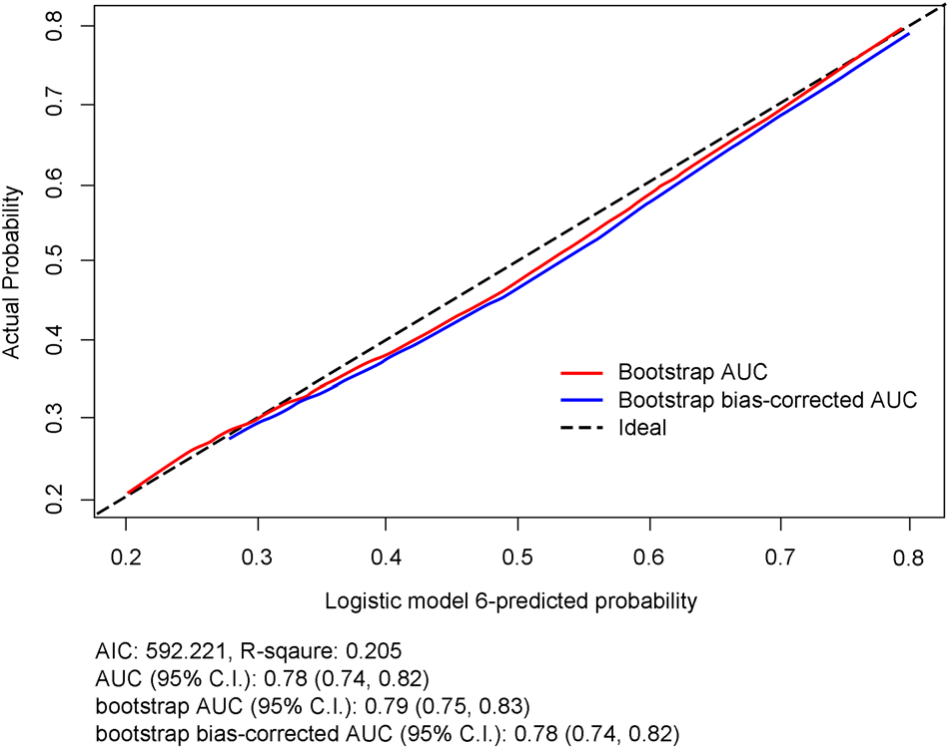
Calibration curve to predict the histologic upgrade of logistic model 6. Notes: The x-axis represents the predicted upgrade risk. The y-axis represents the actual histologic upgrade. The diagonal dotted line represents a perfect prediction by an ideal model. The solid line represents the performance of model 6. The closer the solid line is to the diagonal, the more accurate the prediction.

The results of the multivariable analysis showed a prediction model with the highest Nagelkerke R^2^ index (0.205, range; 0.161–0.205) and the smallest Akaike’s Information Criteria (AIC) (592.221, range; 592.221–627.243) including a biopsy device, nuclear grade, the presence of suspicious LNs on US, and lesion size on mammography and MRI. Underestimation was associated with suspicious axillary LN on US (odds ratio [OR], 12.16; 95% confidence interval [CI], 4.94–29.95; *P* < 0.001) and high nuclear grade (OR, 1.90; 95% CI, 1.24–2.91; *P* = 0.003). Biopsy performed using VAB (OR, 0.42; 95% CI, 0.27–0.65; *P* < 0.001), lesion size <2 cm (OR, 0.45; 95% CI, 0.22–0.90; *P* = 0.021), and non-visible on mammography (OR, 0.41; 95% CI, 0.22–0.76, *P* = 0.002) and lesion size <2 cm (OR, 0.29; 95% CI, 0.09–0.94; *P* = 0.037) or non-visible on MRI (OR, 0.52; 95% CI, 0.28–0.95; *P* = 0.031) were less likely to be underestimated (Table 3 and Supplementary Figs. 1, 2). Six different models that included up to six risk factors were used for adjustment according to various combinations. The combinations of risk factors for each model and their values are shown in the Supplementary Table (online). Among them, Model 6 showed the best performance (Fig. 2).

**Table 3:** Multivariable analysis of factors associated with the histopathologic upgrade to invasive carcinoma.

Variable	Odds ratio (95% CI)	*P*
Device		
CNB	1	
VAB	0.42 (0.27–0.65)	<0.001
Nuclear grade		
Low/Intermediate	1	
High	1.90 (1.24–2.91)	0.003
Suspicious LN on US		
No	1	
Yes	12.16 (4.94–29.95)	<0.001
Lesion size on mammography (cm)		0.002
≥2	1	
<2	0.45 (0.22–0.90)	0.021
Non-visible	0.41 (0.22–0.76)	0.002
Lesion size on MRI (cm)		0.008
≥2	1	
<2	0.29 (0.09–0.94)	0.037
Non- visible	0.52 (0.28–0.95)	0.031

*CI* confidence interval, *CNB* core needle biopsy, *VAB* vacuum-assisted biopsy, *LN* lymph node, *MRI* magnetic resonance imaging, *NME* non-mass enhancement, *US* ultrasonography.

### Performances of the prediction models

**Fig. 3:**
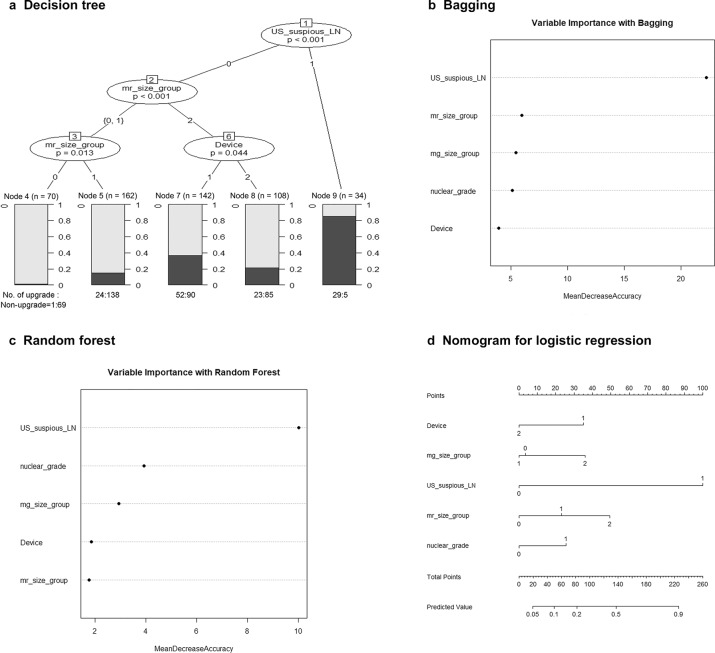
Variable importance graphs of the most important risk factors in descending order and nomogram. **a** decision tree, **b** bagging, and **c** random forest-based machine learning methods and **d** a nomogram for the logistic regression model.

The four prediction models based on logistic regression and three ML techniques showed similar diagnostic performance (Table 4). All four methods showed similar results, with no significant differences in predicting DCIS underestimation. The area under the curves (AUCs) of the four models ranged from 0.66 to 0.78. The three ML methods predicted the risk factors in descending order shown in Fig. 3. All four models reported that the most important risk factor was suspicious axillary LN on US, followed by lesion size on MRI, (except for the random forest technique). In the decision tree technique, only suspicious axillary LNs on US, lesion size on MRI, and biopsy device were significant risk factors, whereas lesion size on mammography and nuclear grade were not used for classification.

**Table 4:** Comparisons of AUCs between four prediction models.

	AUC	95% CI
Logistic regression	0.78	0.74–0.82
Decision tree	0.75	0.56–0.93
Bagging	0.66	0.50–0.83
Random forest	0.75	0.58–0.91

*AUC* area under the receiver operating characteristic curve, *CI* confidence interval.

## Discussion

The rate of underestimation of DCIS by percutaneous biopsy varies from 14% to 43% depending on the imaging guidance and needle gauge^4–6^. The overall DCIS underestimation rate of 25% (161/644) in our study was within this reported range. Of the 161 invasive carcinomas, 73 were revealed as microinvasive carcinoma (11.3%, 73/644). It was a relatively higher incidence than the previously reported number, ~5–10% of cases of DCIS^20^. The higher proportion of cases with microinvasion was likely because the pathology slides are thoroughly read through intensive sampling. The lower proportion of frank invasive cancer compared to that in previous studies may have been because the biopsies were performed by experienced breast radiologists (more than 5 years of experience) in most cases.

Compared to previous studies on DCIS underestimation, our study included all three imaging modalities (e.g., mammography, US, and MRI) and a large number of cases of biopsy-confirmed DCIS (*n* = 644), 161 of which were upgraded to invasive cancer. These features make this the largest study to describe the relationship between imaging findings and underestimation, with more meaningful results obtained through a large number of patients and data from all three imaging modalities. Our results revealed a significantly lower upgrade for mammography than that for US guidance (*P* < 0.001). This is because mammographic guidance is almost always used for lesions that present as calcifications only (without mass), which is associated with the absence of invasion compared to invasive cancer^18^ and, thus, would be consistent with an ultimate diagnosis of DCIS only. Similar to previous studies^4,5,21^, our results revealed that biopsy with VAB (*P* < 0.001), larger sample numbers (*P* = 0.001), and thicker biopsy needles (*P* < 0.001) were associated with a lower occurrence of histopathologic upgrade. Notably, non-visible lesions on US or MRI were less likely to be upgraded to invasive cancer (*P* < 0.001), whereas non-visible lesions on mammography were not significantly associated with the upgrade (*P* = 0.172). We speculate that many lesions could be masked on mammography because most Asian patients have dense breasts.

Our results revealed a relationship between DCIS with invasive components and the presence of suspicious axillary LNs on US (*P* < 0.001). Similar to the previous reports^10,14,22^, clustered ring enhancement of NME (*P* = 0.003) and a washout kinetic pattern at the delayed phase (*P* = 0.002) on MRI were frequently observed in the IDC group. Our results also confirmed that a larger lesion size on mammography or MRI was a predictive factor for the underestimation of invasive cancer^6^. A larger lesion size on mammography was previously reported as an independent predictive factor for invasion, with a cutoff ranging from 20 to 60 mm^2,23^. These features could reflect the assumption that DCIS and invasive cancer are more likely to coexist in large lesions. Moreover, the results of the multivariable analysis revealed high nuclear as a risk factor for underestimation, consistent with previous reports^14,15^.

We applied three ML methods and logistic regression analysis to assess the underestimation risk. ML techniques did not significantly improve the prediction of underestimation. Although the mean size of an upgraded invasive cancer was 3.89 mm in our study, relatively smaller than those reportedly previously^12,15^, the AUCs were relatively good for all models (0.66–0.78).

Our study has some limitations. First, we did not perform an observer study involving multiple readers. Second, most of our patients routinely underwent all three imaging studies in their preoperative evaluations. However, this could differ according to national guidelines or insurance coverage in other countries. Therefore, our results may not be generalizable or reflect all clinical conditions.

In conclusion, the biopsy device, high nuclear grade, the presence of suspicious axillary LN on US, and lesion size >2 cm on mammography or MRI were independent predictors of DCIS underestimation. We observed no significant differences in performance between the conventional prediction and ML models.

## Methods

### Patients

The Institutional Review Board of Samsung Medical Center approved this retrospective study (SMC IRB 2019-12-077-001) and waived the requirement for informed consent due to the retrospective nature of this study. Between January 2012 and September 2018, we reviewed the biopsy database for biopsy-confirmed DCIS at our institution. Once DCIS is diagnosed using biopsy, the standard practice at our institution is to perform mammography, US, and MRI as preoperative imaging workup in all patients. We excluded patients who had not undergone subsequent surgery; without any imaging modality among mammography, US, or MRI; and who had been simultaneously diagnosed with IDC and DCIS in the same breast.

### Biopsy procedure

All needle biopsies were performed using imaging guidance by one of the eight radiologists with 1–26 years of breast imaging experience. US-guided CNB was performed using a 14-or 18-gauge (G) Tru-cut needle with a 22 mm throw (ACECUT, TSK Laboratory, Tokyo, Japan), with a minimum of four cores obtained from each lesion. VABs were performed for small or non-mass lesions or lesions containing calcifications. VABs were also indicated when precise targeting was difficult by the core needle or the in cases in which the results might vary depending on the amount of tissue sample. US-guided VAB was performed using an 8–18-G vacuum-assisted probe (Mammotome, Devicor Endo-Surgery, Cincinnati, OH; Suros, Hologic Inc. Bedford, MA). The needle gauge was determined by lesion size or characteristics and each radiologist’s preference. Stereotactic VAB was performed for microcalcifications that were not visible on US, using an 11-G vacuum-assisted probe (Mammotome, Devicor Endo-Surgery, Cincinnati, OH) and the stereotactic unit of a prone table (Lorad, Hologic Inc., Danbury, CT).

### Data and image analysis

Radiologic variables were collected by reviewing each image retrospectively in consensus by two radiologists (initials blinded) with nine and 14 years of experience in breast imaging who were blinded to the final pathologic outcome. The BI-RADS lexicon was used to describe the mammographic, US, and MRI features^24^. In cases in which the lesion showed no imaging findings, the lesion characteristics were classified as non-visible. The lesion sizes were dichotomized to evaluate the effect of size on the upgrade to invasive cancer by setting a cutoff value of 2 cm, as reported previously^5,6,25^. The following mammography features were evaluated: breast density, lesion characteristics (mass/focal asymmetry, calcifications, combined or non-visible), and lesion size. A mass detected on mammography was evaluated for its shape (oval/round or irregular) and margins (circumscribed/obscured or not circumscribed). Calcifications on mammography were assessed for their morphology (fine linear/branching, fine pleomorphic, coarse heterogeneous/amorphous, or benign appearance) and distribution (linear/segmental or grouped/regional/diffuse).

The following US features were also evaluated: lesion characteristics (mass, non-mass lesion [NML], or non-visible), lesion size, shape (oval/round or irregular), margin (circumscribed or not circumscribed), orientation (parallel or nonparallel), echo pattern (isoechoic, hypoechoic, hyperechoic, or complex/heterogeneous echoic), posterior acoustic features (no posterior feature, enhancement, shadowing, or combined), presence of suspicious axillary LNs, calcifications, and vascularity. An NML was defined as a focal hypoechoic area presenting as a confined asymmetry on two orthogonal planes that could not be characterized as a distinct mass owing to the lack of conspicuous margins or shape that also differed from the surrounding glandular tissue^26^. In addition, the vascularity on color Doppler US was determined according to the number of vessels within or around the lesion and was categorized as low (no flow or only one vessel flow signal observed) or high (more than two vessel flow signals observed)^27^.

Dynamic contrast-enhanced MRI (DCE-MRI) data were reviewed for lesion size, lesion characteristics (mass, NME, or non-visible), lesion morphology (shape, margin, and internal enhancement in mass; distribution and internal enhancement in NME), and time–signal intensity curve pattern. Time–signal intensity curve patterns were categorized based on the presence of washout in the delayed phase. The imaging characteristics in all three modalities were also dichotomized as visible or non-visible to assess whether visibility affected upgrade. The interval between the initial diagnosis and operation was within 1 month in all patients.

All pathologic specimens including biopsy and surgery were read by two experienced breast pathologists (E.Y.C. and S.Y.C. with 20 and 17 years of experience, respectively). After reviewing the postoperative pathology results, the final diagnoses of all lesions were categorized as DCIS or IDC (including microinvasive cancer). We also reviewed the size of invasive cancer in the surgical specimens. The patients’ medical records, including pathological results, were reviewed and data were obtained on age, palpability, and procedural characteristics (guidance methods: US vs. mammography, devices: CNB vs. VAB, needle G, number of core specimens per lesion), nuclear grade, and presence of comedo necrosis from biopsy specimen^28^.

### Statistical analysis

The potential risk factors were statistically compared between the DCIS and IDC groups using Mann–Whitney *U*-tests for continuous variables and chi-square or Fisher’s exact tests for categorical variables. The medians and interquartile ranges (IQRs) were used for continuous variables. First, the risk of the underestimation of invasive breast cancer was analyzed using logistic regression analysis. The associations between all variables and histopathologic upgrade were evaluated using univariable logistic regression analysis. Variables showing a significant association (*P* < 0.05) in the univariable analyses were used as input variables for the multivariable logistic regression analyses. To examine the multicollinearity among these variables, we checked whether the variance inflation factor (VIF) values were 4 or higher. Multicollinearity between variables was considered when building the multivariable models. If multiple multivariable models were built, multivariable logistic regression for multiple models was performed using backward selection. The final prediction model was selected from among the candidate models, in which the lower AIC, the higher Nagelkerke R^2,29,30^, the higher the AUC, and the calibration curve of the actual and predicted probabilities from the model. The AUC was computed from the original sample, from the bootstrap samples with 1000 repetitions. The bias-corrected AUC from the bootstrap samples was also calculated and a calibration plot was presented. The resulting association from the logistic regression was presented with the OR and its 95% CI.

Second, three machine learning methods (decision trees, bagging, and random forests) were applied to determine whether the performance of the prediction of the estimated logistic model was reproduced. Reproducibility was checked for variable importance and AUC values. A brief description of each machine learning technique is provided in the supplementary text (online).

*P* < 0.05 in the two-sided tests were considered statistically significant. All statistical analyses were performed using R (version 3.6.4; R Foundation for Statistical Computing, Vienna, Austria) or SAS (version 9.4, SAS Institute, Cary, NC, USA).

### Reporting Summary

Further information on research design is available in the Nature Research Reporting Summary linked to this article.

## Supplementary information

Supplementary Information

Reporting Summary

## Data Availability

The datasets generated during and/or analysed during the current study are not publicly available due to the institutional regulation but are available from the corresponding author on reasonable request, following ethics committee approval and a data transfer agreement, to guarantee the General Data Protection Regulation. Please contact the corresponding author, E.S.K. (email address: mathilda0330@gmail.com) to request access to the data.
