# Supplementary material for: Ductal carcinoma in situ: a risk prediction model for the underestimation of invasive breast cancer
Source: NPJ Breast Cancer. 2022 Jan 14;8:8. doi: 10.1038/s41523-021-00364-z (PMC8760307; doi:10.1038/s41523-021-00364-z)
Supplement: Supplementary file 1 — Supplementary Information [file 41523_2021_364_MOESM1_ESM.pdf]

Supplementary Files

SUPPLEMENTARY FIGURE LEGENDS

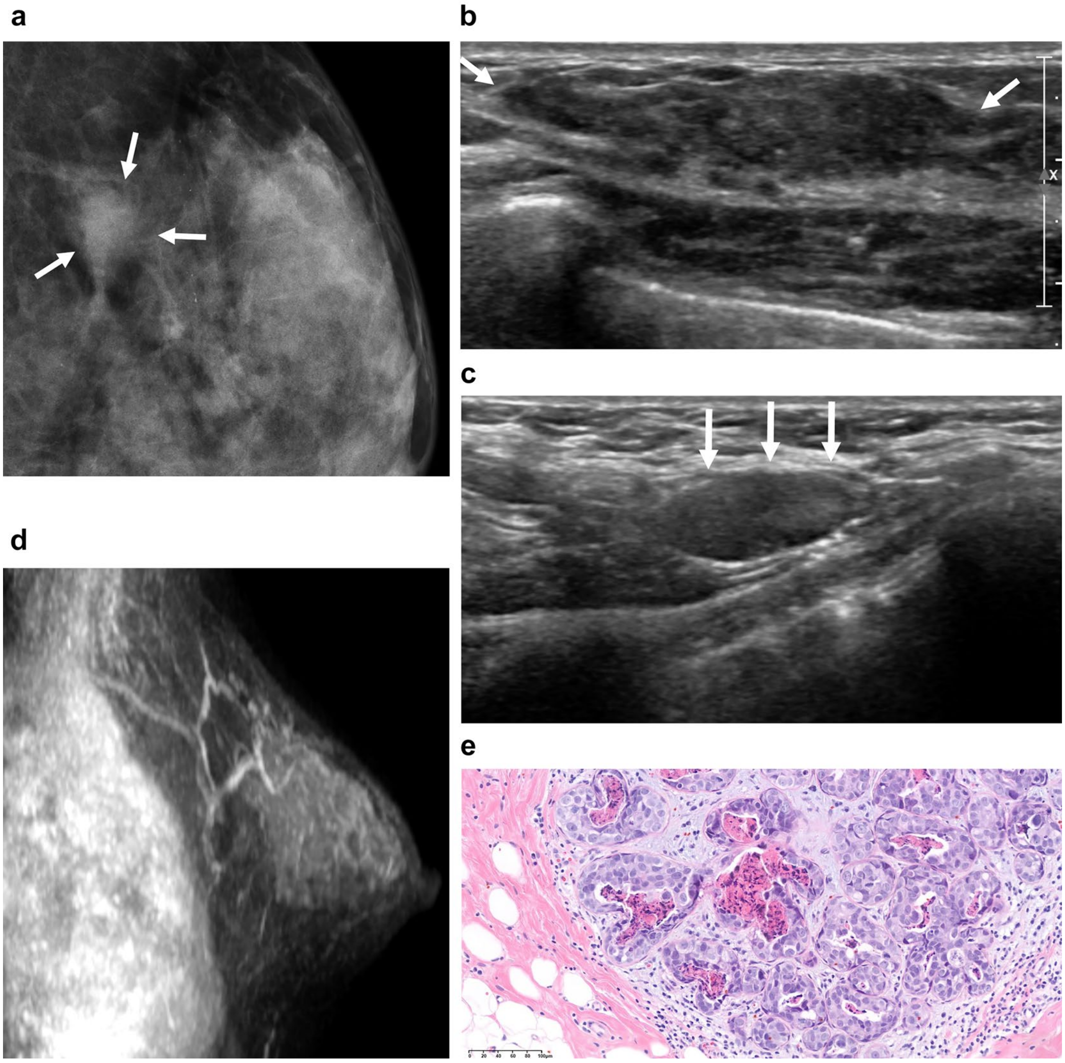

FIG. 1 A 37-year-woman diagnosed with ductal carcinoma in situ by US-guided core needle biopsy. (a) Mediolateral oblique mammography image shows a focal asymmetry in left upper breast (arrows). US image shows a 4.3cm hypoechoic mass in left 10 o'clock (b) and suspicious axillary LN (c) (arrows). (d) MRI shows a 6.7 cm segemental non-mass enhancement in left upper outer breast (arrows). (e) H&E staning of core needle biopsy shows comedo type high grade DCIS. There is central comedo necrosis (x100). Subsequent surgery revealed 3mm of invasive ductal carcinoma and this is a representative case of upgrade.

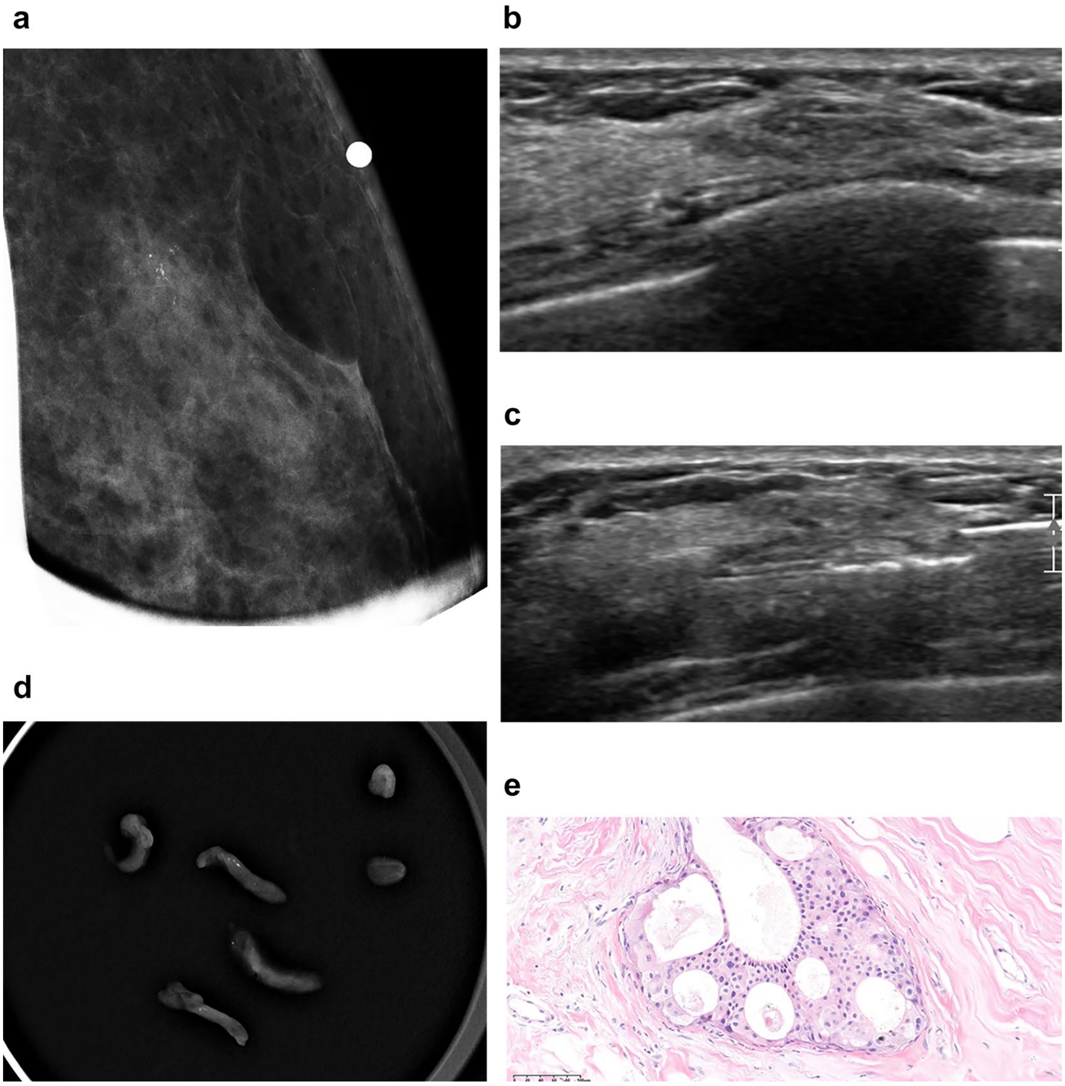

Fig 2 A 57-year-old woman diagnosed with ductal carcinoma in situ by US-guided vacuum assiusted biopsy. (a) Craniocaudal mammography image shows fine pleomorphic microcalcifications in left outer breast. (b) US image shows a 1.4 cm isoechoic mass in left 3 o'clock. (c) After US-guided vaccum-assisted biopsy on (b), Specimen mammography image shows microcalcifications in the biopsy cores. (d) MRI shows a 1.5cm focal non-mass enhancement in left far outer breast. (e) H&E staning of vaccum-assisted biopsy shows cribriform type intermidiate grade DCIS. The nuclei are slightly enlarged, and show moderate size variation (x100). Final pathology revealed only DCIS without invasive foci.

Supplementary Table. Result of 6 models with different combination of risk factors Using Bootstrap Samples with 1,000 Repetitions.

	AIC	R^2	AUC form original sample	AUC from bootstrap sample	Bias-corrected AUC form bootstrap sample
Model 1†	627.243	0.161	0.74	0.74	0.72
Model 2†	619.409	0.163	0.74	0.74	0.73
Model 3†	599.939	0.196	0.78	0.78	0.77
Model 4†	627.243	0.161	0.74	0.74	0.72
Model 5†	619.409	0.163	0.74	0.74	0.73
Model 6†	592.221	0.205	0.78	0.79	0.78

† Results from different combination of risk factors.

Model 1: Palpability, mammography characteristics, suspicious LN on US, nuclear grade.

Model 2: Palpability, suspicious LN on US , dichotomized MRI characteristics, nuclear grade.

Model 3: Palpability, mammography lesion size, suspicious LN on US, MR lesion size, nuclear grade.

Model 4: Palpability, mammography characteristics, suspicious LN on US, nuclear grade.

Model 5: Palpability, suspicious LN on US, dichotomized MRI characteristics, nuclear grade.

Model 6: Device, mammogrphy lesion size, suspicious LN on US, MRI lesion size, nuclear grade.

AIC Akaike's Information Criteria, R^2 Nagelkerke R^2 index, *AUC* area under the receiver operating characteristic curve.

SUPPLEMENTARY METHODS

Random forest

Random Forest, as an ensemble of decision trees, can solve both regression and classification problems with large data sets. Random Forest is highly scalable to any number of dimensions and has generally quite acceptable performances. The number of trees to grow was set at 500 in the R package[1].

Decision tree

A decision tree classifier utilizes a tree structure to model relationships among the features and the potential outcomes. A standard classification and regression tree algorithm is used to select the split predictor that maximizes the split-criterion gain over all possible splits of all predictors. Finding the optimal size of the tree helps to improve the predictive accuracy through the reduction of overfitting. We optimized tree depth using an R-package pruning technique that merges leaves on the same tree branch[2].

Bagging

Bagging is a method for improving results of machine learning classification algorithms. This method was formulated by Breiman and its name was deduced from the phrase "bootstrap aggregating." Bagging is usually applied to decision tree methods, though it can be used with other classifiers. In summary, bagged tree resamples the training dataset several times (bootstrapping) and builds a decision tree model from each; it then aggregate these models together for a final classifier. In our study, the analysis for bagging was implemented with the ipred package[3].

REFERENCES

1. Ho, T. K. The random subspace method for constructing decision forests. *IEEE Trans. Pattern Anal. Mach. Intell.* **20**, 832-844 (1998).
2. Breiman, L., Friedman, J. H., Olshen, R. A. & Stone, C. J. *Classification and regression trees* (Routledge, 2017).
3. Mohebian, M. R., Marateb, H. R., Mansourian, M., Mananas, M. A. & Mokarian, F. A Hybrid Computer-aided-diagnosis System for Prediction of Breast Cancer Recurrence (HPBCR) Using Optimized Ensemble Learning. *Comput. Struct. Biotec.* **15**, 75-85 (2017).
