# Supplementary material for: Ductal carcinoma in situ: a risk prediction model for the underestimation of invasive breast cancer
Source: NPJ Breast Cancer. 2022 Jan 14;8:8. doi: 10.1038/s41523-021-00364-z (PMC8760307; doi:10.1038/s41523-021-00364-z)
Supplement: Supplementary file 2 — Reporting Summary [file 41523_2021_364_MOESM2_ESM.pdf]

nature portfolio

NPJBCANCER-01380R
Corresponding author(s): Eun Sook Ko
Last updated by author(s): Nov 15, 2021

Reporting Summary

Nature Portfolio wishes to improve the reproducibility of the work that we publish. This form provides structure for consistency and transparency in reporting. For further information on Nature Portfolio policies, see our Editorial Policies and the Editorial Policy Checklist.

Statistics

For all statistical analyses, confirm that the following items are present in the figure legend, table legend, main text, or Methods section.

n/a	Confirmed	
☐	☒	The exact sample size (*n*) for each experimental group/condition, given as a discrete number and unit of measurement
☐	☒	A statement on whether measurements were taken from distinct samples or whether the same sample was measured repeatedly
☐	☒	The statistical test(s) used AND whether they are one- or two-sided<br>*Only common tests should be described solely by name; describe more complex techniques in the Methods section.*
☐	☒	A description of all covariates tested
☐	☒	A description of any assumptions or corrections, such as tests of normality and adjustment for multiple comparisons
☐	☒	A full description of the statistical parameters including central tendency (e.g. means) or other basic estimates (e.g. regression coefficient) AND variation (e.g. standard deviation) or associated estimates of uncertainty (e.g. confidence intervals)
☒	☐	For null hypothesis testing, the test statistic (e.g. *F*, *t*, *r*) with confidence intervals, effect sizes, degrees of freedom and *P* value noted<br>*Give P values as exact values whenever suitable.*
☒	☐	For Bayesian analysis, information on the choice of priors and Markov chain Monte Carlo settings
☒	☐	For hierarchical and complex designs, identification of the appropriate level for tests and full reporting of outcomes
☒	☐	Estimates of effect sizes (e.g. Cohen's *d*, Pearson's *r*), indicating how they were calculated

Our web collection on statistics for biologists contains articles on many of the points above.

Software and code

Policy information about availability of computer code

Data collection	NA
Data analysis	All statistical analyses were performed using R (version 3.6.4; R Foundation for Statistical Computing, Vienna, Austria) or SAS (version 9.4, SAS Institute, Cary, NC, USA).

For manuscripts utilizing custom algorithms or software that are central to the research but not yet described in published literature, software must be made available to editors and reviewers. We strongly encourage code deposition in a community repository (e.g. GitHub). See the Nature Portfolio guidelines for submitting code & software for further information.

Data

Policy information about availability of data

All manuscripts must include a data availability statement. This statement should provide the following information, where applicable:
- Accession codes, unique identifiers, or web links for publicly available datasets
- A description of any restrictions on data availability
- For clinical datasets or third party data, please ensure that the statement adheres to our policy

The datasets that support the findings of this study are not publicly available but will be made available upon reasonable request, following ethics committee approval and a data transfer agreement, to guarantee the General Data Protection Regulation. Please contact the corresponding author, E.S.K (email address: mathilda0330@gmail.com) to request access to the data.

Field-specific reporting

Please select the one below that is the best fit for your research. If you are not sure, read the appropriate sections before making your selection.

☒ Life sciences ☐ Behavioural & social sciences ☐ Ecological, evolutionary & environmental sciences

For a reference copy of the document with all sections, see nature.com/documents/nr-reporting-summary-flat.pdf

Life sciences study design

All studies must disclose on these points even when the disclosure is negative.

Sample size	Between January 2012 and September 2018, we reviewed the biopsy database for biopsy-confirmed DCIS at our institution.
Data exclusions	We excluded patients who had not undergone subsequent surgery; without any imaging modality among mammography, US, or MRI; and who had been simultaneously diagnosed with IDC and DCIS in the same breast.
Replication	NA
Randomization	NA
Blinding	Radiologic variables were collected by reviewing each image retrospectively in consensus by two radiologists (initials blinded) with nine and 14 years of experience in breast imaging who were blinded to the final pathologic outcome.

Reporting for specific materials, systems and methods

We require information from authors about some types of materials, experimental systems and methods used in many studies. Here, indicate whether each material, system or method listed is relevant to your study. If you are not sure if a list item applies to your research, read the appropriate section before selecting a response.

Materials & experimental systems

n/a	Involved in the study	
☒	☐	Antibodies
☒	☐	Eukaryotic cell lines
☒	☐	Palaeontology and archaeology
☒	☐	Animals and other organisms
☐	☒	Human research participants
☒	☐	Clinical data
☒	☐	Dual use research of concern

Methods

n/a	Involved in the study	
☒	☐	ChIP-seq
☒	☐	Flow cytometry
☒	☐	MRI-based neuroimaging

Human research participants

Policy information about studies involving human research participants

Population characteristics	Between January 2012 and September 2018, we reviewed the biopsy database for biopsy-confirmed DCIS at our institution. Once DCIS is diagnosed using biopsy, the standard practice at our institution is to perform mammography, US, and MRI as preoperative imaging workup in all patients.
Recruitment	Between January 2012 and September 2018, we reviewed the biopsy database for biopsy-confirmed DCIS at our institution.
Ethics oversight	The Institutional Review Board of Samsung Medical Center approved this retrospective study (SMC IRB 2019-12-077-001) and waived the requirement for informed consent due to retrospective nature of this study.

Note that full information on the approval of the study protocol must also be provided in the manuscript.
